# Deoxyshikonin isolated from *Arnebia euchroma* inhibits colorectal cancer by down-regulating the PI3K/Akt/mTOR pathway

**DOI:** 10.1080/13880209.2019.1626447

**Published:** 2019-06-23

**Authors:** Yuzhen Zhu, Yu Zhong, Xun Long, Zhu Zhu, Yu Zhou, Hua Ye, Xiaobin Zeng, Xuebao Zheng

**Affiliations:** a Guangdong Key Laboratory for Research and Development of Natural Drugs, Guangdong Medical University, Zhanjiang, China;; b Analysis Center of Guangdong Medical University, Zhanjiang, China;; c The Third People’s Hospital of Bijie, Bijie, China;; d Sino-American Cancer Research Institute, Guangdong Medical University, Dongguan, China;; e Department of Gastroenterology, Affiliated Hospital of Guangdong Medical University, Zhanjiang, China;; f Center Lab of Longhua Branch, Shenzhen People’s Hospital, 2nd Clinical Medical College of Jinan University, Shenzhen, China;; g Department of Infectious disease, Shenzhen People’s Hospital, 2nd Clinical Medical College of Jinan University, Shenzhen, China;; h Mathematical Engineering Academy of Chinese Medicine, Guangzhou University of Chinese Medicine, Guangzhou, China

**Keywords:** Shikonin, apoptosis, proliferation, cell cycle

## Abstract

**Context:** Shikonins, a series of natural occurring naphthoquinones extracted from *Arnebia euchroma* (Royle) Jonst. (Boraginaceae), have antitumor activities and low toxicity.

**Objective:** To illuminate potential activity and mechanism of shikonins against colorectal cancer (CRC).

**Materials and methods:** Five shikonins were isolated from *A. euchroma*, and elucidated by extensive spectroscopic analysis. Anti-proliferative activities of shikonins (0–100 μg/mL) on human colorectal cells were evaluated by MTT and CCK-8 for 24 or 48 h. Cell apoptosis and cycle distribution were examined by FCM analysis. The expression of PI3K/Akt/mTOR pathway mRNAs and proteins was analysed by RT-PCR and Western blot, respectively. Cell viability, cell apoptosis, cell cycle and protein expression were measured, when co-treated with PI3K/Akt/mTOR pathway inhibitors. The *in vivo* activity of deoxyshikonin was evaluated using xenograft tumour model.

**Results:** Deoxyshikonin and another four shikonins were isolated and identified. Deoxyshikonin exhibited anti-proliferative activity with IC_50_ of 10.97 μM against HT29 cells. Moreover, the percentage of early apoptotic cells and G0/G1 cells increased from 1 to 29% and 44 to 67% with 0–50 μg/mL deoxyshikonin, respectively. Deoxyshikonin also down-regulated the expression of PI3K, p-PI3K, Akt, p-Akt308 and mTOR proteins in HT29 and DLD-1 cells. Moreover, LY294002, NVP-BEZ235 and MK-2206 can make deoxyshikonin more cell proliferation inhibited, cell cycle arrested at G0/G1 and apoptosis promoted. *In vivo* study, the weight of tumour tissues at deoxyshikonin groups was significantly reduced compared with the control group, and PI3K, p-PI3K, Akt, p-Akt308 and mTOR expression was decreased.

**Discussion and conclusions:** We can conclude that deoxyshikonin isolated from *Arnebia euchroma* inhibited CRC through the PI3K/Akt/mTOR pathway.

## Introduction

Colorectal cancer (CRC) is the fourth most fatal cancer in worldwide, and the incidence and prevalence of CRC are gradually rising (Turati et al. 2017). The treatment options include surgery at an early stage; it is combined with radiotherapy and chemotherapy in the late stage. Nowadays, with the help of these chemotherapeutic agents including 5-fluorouracil, capecitabine, irinotecan, oxaliplatin, bevacizumab and dabrafenib, the 5-year survival rate has increased for patients who have colon and rectal cancers (Hochster et al. 1994; van Hazel et al. 2009). However, the side effects of systemic therapy such as resistance to cytotoxic and drug resistance have a great impact on the quality of patients’ lives (Longley et al. 2003; Kim and Park 2016; McQuade et al. 2017). Hence, the clinical treatment of CRC requires a drug with higher efficiency and fewer side effects. For many years, natural products have provided an efficient resource for the discovery of potential antitumour therapeutic agents. Active antitumour substances from traditional Chinese medicines and natural products are widely used in clinical cancer treatment, such as vinblastine (Lukesh et al. 2017), taxol (Ganz et al. 2017), harringtonine (Klanova et al. 2016), etoposide (Yuan et al. 2017), arsenic trioxide (Kutny et al. 2017) and elemene (Yu et al. 2008). Therefore, a novel effective Chinese medicine monomer with high efficiency and low toxicity for CRC has great potential for development of medicine.

Shikonin, a natural occurring naphthoquinone compound, is the primary component of *Arnebia euchroma* (Royle) Jonst (Boraginaceae). In the pharmaceutical and medical field, shikonin currently has aroused growing attention for immune regulation (Li et al. 2013), bacteria inhibition (Kuo et al. 2004; Zhao et al. 2017) and antitumour activity (Ahn et al. 2013; Lin et al. 2015; Jeung et al. 2016). Importantly, shikonin has shown potent anti-proliferative and apoptosis activities against multiple tumour cells in recent years, including lung (Jeung et al. 2016) and pancreatic cancer cells (Lin et al. 2015) and HaCaT cells (Ahn et al. 2013). However, the relatively low Ki values of shikonin would have a high-risk potential to cause possible toxicity, especially drug–drug or food–drug interactions based on the potent inhibition of CYP enzymes (Tang et al. 2017). Hence, there has been increased interest in the potent shikonin derivatives with lower toxicity and stronger antitumour activities (Lin et al. 2015; Lu et al. 2015). Interestingly, no haematological or non-haematological toxicity is observed in a rat model with a dose of up to 800 mg/kg shikonin derivative daily for 6 months (Su et al. 2014). Furthermore, shikonin derivatives bind well to tubulin in colchicine to promote tumour cell apoptosis (Qiu, Wang, et al. 2017), suppress nuclear localization of STAT3 to inhibit breast cancer cells (Qiu, Zhu, et al. 2017), or reduce tumour growth by inhibiting medullary thyroid carcinoma cell proliferation and inducing apoptosis (Hasenoehrl et al. 2017). In mechanistic research, shikonin derivative (β-hydroxyisovaleryl-shikonin) promoted cervical cancer cell apoptosis via PI3K/Akt/mTOR pathway (Lu et al. 2015). However, the antitumour activity of these shikonin derivatives on human colon cancer and their molecular mechanism remain unclear.

Therefore, this study focused on the extraction and identification effective shikonin derivatives of *Arnebia euchroma*. Their effects and their potential mechanisms on CRC were also explored.

## Materials and methods

### Extraction and isolation

The roots of *A. euchroma* were purchased in September 2015 in Haozhou, Anhui Province, China, and subjected to taxonomic identification by Xiaobin Zeng with voucher specimens (no. 161001) deposited at Center Lab of Longhua Branch, Shenzhen People’s Hospital, 2nd Clinical Medical College of Jinan University in Shenzhen, China. The roots of *A. euchroma* (7.5 kg) were extracted three times with 95% ethanol. The solvent was removed under vacuum to yield the crude extract (1150 g). The crude extract was resuspended in water and partitioned with chloroform (3 L × 3), ethyl acetate (3 L × 3) and water-saturated *n*-butanol (3 L × 3) gradually to afford 178.21, 62.01 and 254.35 g of dried organic extracts, respectively.

Bioactivity-guided fractionation was used for the isolation work. On the basis of the bioactive results of the extracts, the chloroform fraction with the most potential activity was fractionated over a silica gel (200–300 mesh) column eluting with a gradually amount of MeOH in CHCl_3_ to give five fractions. The CHCl_3_–MeOH (40:1) elution (25.0 g) was further purified by a Sephadex LH-20 column (6 × 88 cm, 680 g; solvent system: CHCl_3_–MeOH, 7:3) to obtain three major fractions, 1a–1c. Fraction 1a (15.0 g) was applied to a preparative HPLC with 89% methanol (containing 0.1% CF_3_COOH, pH 3.0) to give fractions 1a1–1a3, compounds **4** (2052.03 mg) and **5** (3570.17 mg), respectively. Fraction 1a3 (1205.2 mg) was further purified by preparative HPLC (MeOH–H_2_O, 86:14, containing 0.1% CF_3_COOH, pH 3.0) at 8 mL/min for 58 min to obtain **3** (848.27 mg). Also, compounds **1** (234.0 mg) and **2** (1122.15 mg) were purified by preparative HPLC (83% MeOH and 83% MeOH, containing 0.1% CF_3_COOH, pH 3.0) at 8 mL/min from fraction 1a1 (512.1 mg) and fraction 1a2 (1421.1 mg), respectively.

### Cell culture and treatments

Human colon cancer cell lines including HT29, DLD-1, HCT116 and Caco-2, were purchased from the ATCC (PA) (Manassas, USA). These cells were grown in RPMI 1640 (Invitrogen, Carlsbad, CA) supplemented with 10% (v/v) foetal bovine serum (Invitrogen, Carlsbad, CA) and 1% penicillin–streptomycin (Invitrogen, Carlsbad, CA) at 37 ± 0.5 °C in a humidified incubator (Thermo Scientific, Waltham, MA). Shikonin derivatives were dissolved in DMSO (Sigma-Aldrich, St. Louis, MO) and further diluted in culture medium. Cells were treated with various concentrations of shikonin derivatives (0–100 μg/mL) for different time. Then, cells were collected and subjected to further analysis.

### Methyl thiazolyl tetrazolium (MTT) assay

HT29 cells in the exponential growth phase were seed onto 96-well plates at a density of 5 × 10^3^ cells/well. After 24 h, the medium was replaced with fresh medium and then adhered cells were treated with different concentrations of shikonin derivatives (0, 6.25, 12.5, 25, 50 and 100 μg/mL) for 24 or 48 h. Next, MTT solution (2 mg/mL in PBS, Sigma-Aldrich, St. Louis, MO) was added to each well and incubated the cells for an additional 4 h. After removing the supernatants, added 50 μL DMSO to dissolve the tetrazolium salt and measure its optical density (OD) using Multiskan Spectrum Microplate Reader (Thermo Labsystems, Milan, Italy) at 490 nm. The experiment was repeated three times. Cytotoxicity of shikonin derivatives were assessed as described previously, and calculated according to the following formula:

$$\text{Inhibitory}\;\text{rate}\;\left(\%\right)\;=\;\left({\text{OD}}_{\text{Control}}-{\;\text{OD}}_{\text{Sample}}\right)/\left({\text{OD}}_{\text{Control}}-{\;\text{OD}}_{\text{Blank}}\right)\times 100\%$$

### Cell counting kit-8 (CCK-8) assay

Human colorectal cells including Caco-2, HCT116, DLD-1 and HT29 cells seeded onto 96-well plates (5 × 10^3^ cells per well). After incubation for 24 h, shikonin derivatives (0, 6.25, 12.5, 25, 50 and 100 μg/mL) were added to the medium for additional 48 h. Then, cells were incubated with CCK-8 solution (10 μL, Dojindo Laboratories, Kumamoto, Japan) for 2 h. The absorbance was measured at 450 nm by Multiskan Spectrum Microplate Reader (Thermo Labsystems, Milan, Italy). The experiment was done in triplicate. The inhibitory rate of cell proliferation was calculated by the following formula:

$$\text{Inhibitory}\;\text{rate}\;\left(\%\right)\;=1\;-\;\left({\text{OD}}_{\text{Sample}}-{\;\text{OD}}_{\text{Blank}}\right)/\left({\text{OD}}_{\text{Control}}-{\;\text{OD}}_{\text{Blank}}\right)\times 100.$$

### Apoptosis detection assay

HT29 cells were exposed to different concentrations of deoxyshikonin (0, 25, 50 and 100 μg/mL) at a fixed time (24 or 48 h) in six-well culture plates (5 × 10^5^ cells per well). Then, we harvested the cells and supernatant, washed in PBS and resuspended 1 × 10^6^ cells/mL in 1× binding buffer. Next, FITC-conjugated Annexin V and propidium iodide (BD Biosciences Pharmingen, Franklin Lakes, NJ) were added to the 100 μL cell suspension. Finally, the samples were measured with a BD FACSCanto™ II (BD Biosciences, San Jose, CA) after incubation in the dark for 15 min at room temperature. Annexin V single positive represented the cells in the early apoptotic stage, while both Annexin V and PI positive represented the cells in the last stage of apoptosis, and PI single positive cells represented necrotic cells. The experiment was performed three times.

### Cell cycle analysis assay

Briefly, HT29 cells were trypsinized with 0.25% trypsin EDTA at room temperature after pretreatment with various concentrations of deoxyshikonin at different times and washed three times in buffer solution. Then, deoxyshikonin-induced cell cycle distribution was detected by Cycle test Plus DNA reagent kit (BD Biosciences Pharmingen, Franklin Lakes, NJ). The percentages of cells in G0/G1, S and G2/M phases were detected by flow cytometry.

### Western blotting

HT29 cells (5 × 10^5^) were treated with deoxyshikonin in a six-well culture plate. After 48 h, we harvested the cells on ice and cleavage in RIPA lysate with 1 mM PMSF and phosphatase inhibitor for 30 min. Then, the cells were centrifuged at 12,000 rpm for 30 min at 4 °C. Next, we placed the sample on a vortex mixer, and heated in a 90 °C bath for 10 min. For tumour tissues, we placed the small pieces in lysis buffer and sonicated for 8–10 s. Then, the samples were centrifuged at 12,000 rpm for 30 min at 4 °C. After preparing the protein samples, we performed the Western blotting according to standard protocols. Briefly, the PVDF membrane containing the target protein was washed with TBST solution, incubated with 5% nonfat milk solution and primary antibodies: PI3K, p-PI3K, Akt, p-Akt308, mTOR and β-actin (Cell Signaling Technology, Boston, MA). The suspensions were gently shaken overnight at 4 °C. Then, we add HRP-conjugated polymer-tagged secondary antibodies (Abcam, Cambridge, MA) with diluted in blocking solution and incubated for 1 h on a shaker at room temperature. Lastly, the protein bands were captured using Western blotting detection system (DNR Bio-Imaging Systems, Jerusalem, Israel). Signal intensity was quantified by densitometry with the Gel-pro Analyzer (Media Cybernetics, Rockville, MD). All experiments were done in triplicate.

### Quantitative real-time PCR

Total RNA was extracted according to the total RNA Extraction Kit procedure (Omega, GA) and subjected to reverse transcription using reverse transcriptase (Roche, Basel, Switzerland) and SYBR Premix ExTaq kit (Takara, Dalian, China). The gene-specific primers were as follows: AKT forward, 5′-CTTGCTTTCAGGGCTGCTCA-3′ and reverse, 5′-TACACGTGCTGCCACACGATAC-3′; PI3K forward, 5′-CTGTCAATCGGTGACTGTGTGG-3′ and reverse, 5′-AAACAGGTCAATGGCTGCATCATA-3′; mTOR forward, 5′-AGAAACTGCACGTCAGCA CCA-3′ and reverse, 5′-CCATTCCAGCCAGTCATCTTTG-3′; β-actin forward, 5′-GCTTCTAGGCGGACTGTTAC-3′ and reverse, 5′-CCATGCCAATGTTGTCTCTT-3′. Samples were amplified by the Applied Biosystems 7500 Real-Time PCR System (Life Technologies Corporation, Carlsbad, CA). The conditions of qRT-PCR were as follows: 95 °C initial denaturation for 1 min, followed by 40 cycles of denaturation (10 s at 95 °C), annealing (40 s at 60 °C). Each sample was amplified in triplicate. The results were calculated using the 2^–ΔΔCt^ method.

### Inhibition of PI3K/Akt/mTOR pathway

To study the effects of inhibitors on antitumour function of deoxyshikonin, HT29 cells were pretreated with LY294002 (10 mM, Selleck Chemicals, Houston, TX), NVP-BEZ235 (1 mM, Selleck Chemicals, Houston, TX) or MK-2206 (1 mM, Selleck Chemicals, Houston, TX) for 30 min, then co-treated with 50 μg/mL deoxyshikonin for further 48 h. Then, cell viability, cell apoptosis, cell cycle arrest and expression of PI3K/Akt/mTOR proteins were measured in terms of the above experimental methods.

### Tumour xenograft in athymic BALB/c nude mice

A total of 20 male BALB/c nude mice with body weights of 10–14 g, were purchased from Hunan SJA Laboratory Animal Limited Company and maintained in a specific pathogen-free environment. Animal experiments were approved by the Guangdong Medical University Institutional Animal Care and Use Committee. After 2 weeks, mice were injected with DLD-1 cells (1 × 10^7^ cell in a volume of 0.2 mL) into the subcutaneous tissue of the right auxiliary region. The volume of the tumour was measured every other day by caliper and calculated by the following formula: (length × width × width)/2. When tumours reached a volume of 62.5 mm^3^, mice were divided randomly into the following four groups and given different drugs by intraperitoneal injection for a total of 13 days: control, DMSO (1% DMSO, every two days), deoxyshikonin (20 mg/kg in 1% DMSO, every two days), irinotecan (66.7 mg/kg, every four days, Qilu Pharmaceutical Co. Ltd, Hainan, China). On day 13, all mice were sacrificed. The tumours were segregated, measured and stored in liquid nitrogen for later use.

### Haematoxylin/eosin (H&E) staining

Tumours were collected, fixed in 10% formalin, and embedded in paraffin. Tissue sections (5 μm in thickness) were prepared in light of standard protocols for H&E staining. After staining, the tissue slices were viewed under microscopy at ×200 magnification with the Olympus DP controller software program (Tokyo, Japan).

### Statistical analysis

All the experiments were performed independently at least three times. The results were analysed using GraphPad Prism version 6.0 (GraphPad Software, La Jolla, CA) to perform one-way ANOVA. A *p* value less than 0.05 was considered statistically significant.

## Results

### Isolates

Deoxyshikonin (**1**) (purity: 95.3%) was a red amorphous powder; [α]_25_^D^ 0 (c 0.20, CHCl_3_); ^1^H NMR (CDCl_3_, 400 MHz) data: 6.85 (1H, s, H-3), 7.20 (2H, s, H-7, 8), 12.47 (1H, s, 9-OH), 12.63 (1H, s, 6-OH), 2.64 (2H, t, *J* = 6.8 Hz, H-1′), 2.31 (2H, m, H-2′), 5.15 (1H, m, H-3′), 1.60 (3H, s, 5′-CH_3_), 1.70 (3H, s, 6′-CH_3_); ^13^C NMR (CDCl_3_, 100 MHz) data: 182.4 (C-1), 150.9 (C-2), 133.9 (C-3), 182.3 (C-4), 111.4 (C-5), 161.8 (C-6), 130.3 (C-7), 130.6 (C-8), 162.5 (C-9), 111.2 (C-10), 29.1 (C-1′), 26.0 (C-2′) 121.8 (C-3′), 133.1 (C-4′), 17.2 (C-5′) and 25.1 (C-6′).

Acetylshikonin (**2**) (purity: 96.1%) was a red amorphous powder; [α]_25_^D^ +151.2 (c 0.20, CHCl_3_); ^1^H NMR (CDCl_3_, 400 MHz) data: 6.99 (1H, s, H-3), 7.18 (2H, s, H-6, 7), 12.42 (1H, s, 6-OH), 12.58 (1H, s, 5-OH), 6.03 (1H, dd, *J* = 7.2, 4.4 Hz, H-1′), 2.46 (1H, m, H-2′), 2.62 (1H, m, H-2′), 5.12 (1H, t, *J* = 7.6 Hz, H-3′), 1.69 (3H, s, 5′-CH_3_), 1.58 (3H, s, 6′-CH_3_), 2.14 (3H, s, H-2″); ^13^C NMR (CDCl_3_, 100 MHz) data: 178.1 (C-1), 148.2 (C-2), 136.1 (C-3), 176.6 (C-4), 111.6 (C-5), 169.7 (C-6), 131.5 (C-7), 132.7 (C-8), 167.0 (C-9), 111.8 (C-10), 69.5 (C-1′), 32.8 (C-2′), 117.6 (C-3′), 132.9 (C-4′), 17.9 (C-5′), 25.7 (C-6′), 167.6 (C-1″) and 20.9 (C-2″).

Isobutyrylshikonin (**3**) (purity: 94.1%) was a red amorphous powder; [α]_25_^D^ +166.3 (c 0.20, CHCl_3_); ^1^H NMR (CDCl_3_, 400 MHz) data: 1.17 (3H, d, *J* = 4.4 Hz, 3″, 4″-CH_3_), 1.20 (3H, d, *J* = 4.0 Hz, 6′-CH_3_), 1.56 (3H, s, 6′-CH_3_), 1.67 (3H, s, 5′-CH_3_), 2.46 (1H, m, H-2′), 2.60 (2H, m, H-2′, 2″), 5.10 (1H, t, *J* = 6.0 Hz, CH = C-), 6.01 (1H, m, H-1′), 6.95 (1H, d, *J* = 0.8 Hz, H-3), 7.16 (2H, s, H-6, 7), 12.40 (1H, s, 6-OH), 12.56 (1H, s, 5-OH); ^13^C NMR (CDCl_3_, 100 MHz) data: 177.7 (C-1), 148.0 (C-2), 130.8 (C-3), 176.2 (C-4), 166.8 (C-5), 132.1 (C-6), 132.3 (C-7), 166.3 (C-8), 111.4 (C-9), 111.1 (C-10), 68.5 (C-1′), 33.5 (C-2′), 117.2 (C-3′), 135.2 (C-4′), 25.2 (C-5′), 17.4 (C-6′), 175.1 (C-1″), 32.4 (C-2″), 18.4 (C-3″) and 18.3 (C-4″).

β,β′-Dimethylacrylshikonin (**4**) (purity: 94.4%) was a red amorphous powder; [α]_25_^D^ +154.2 (c 0.20, CHCl_3_); ^1^H NMR (CDCl_3_, 400 MHz) data: 6.98 (1H, d, *J* = 0.8 Hz, H-3), 12.43 (1H, s, 5-OH), 7.18 (2H, s, H-6 and 7), 12.60 (1H, s, 8-OH), 6.02 (1H, dd, *J* = 6.8, 4.8 Hz, H-1′), 2.61 (1H, m, H-2′), 5.15 (1H, t, *J* = 7.6 Hz, H-3′), 1.67 (3H, s, H-5′), 1.58 (3H, s, H-6′), 2.48 (1H, m), 3.78 (1H, d, *J* = 1.2 Hz), 2.16 (3H, s, H-4″), 1.94 (3H, s, H-5″); ^13^C NMR (CDCl_3_, 100 MHz) data: 177.5 (C-1), 149.0 (C-2), 131.6 (C-3), 179.0 (C-4), 166.3 (C-5), 132.5 (C-6), 132.6 (C-7), 166.9 (C-8), 111.9 (C-9), 111.6 (C-10), 68.6 (C-1′), 32.9 (C-2′), 118.0 (C-3′), 135.8 (C-4′), 25.7 (C-5′), 17.9 (C-6′), 165.3 (C-1″), 115.3 (C-2″), 158.9 (C-3″), 20.4 (C-4″) and 29.7 (C-5″).

Isovalerylshikonin (**5**) (purity: 95.3%) was a red amorphous powder; [α]_25_^D^ +157.2 (c 0.20, CHCl_3_); ^1^H NMR (CDCl_3_, 400 MHz) data: 6.99 (1H, d, *J* = 0.4 Hz, H-3), 7.19 (2H, d, *J* = 8 Hz, H-7, 8), 12.42 (1H, s, H-6), 12.60 (1H, s, H-9), 5.13 (1H, t, *J* = 7.2 Hz, H-1′), 2.60 (1H, m, H-2′), 2.47 (1H, m, H-2′), 6.05 (1H, m, H-3′), 1.69 (3H, s, H-5′), 1.58 (3H, s, H-6′), 2.27 (2H, dd, *J* = 7.6, 3.2 Hz, H-2″), 2.13 (1H, m, H-3″), 0.95 (6H, m, H-4″, 5″); ^13^C NMR (CDCl_3_, 100 MHz) data: 178.7 (C-1), 148.5 (C-2), 131.4 (C-3), 178.2 (C-4), 167.0 (C-5), 132.7 (C-6), 132.9 (C-7), 167.5 (C-8), 111.6 (C-9), 111.7 (C-10), 69.1 (C-1′), 32.9 (C-2′), 117.9 (C-3′), 136.0 (C-4′), 25.7 (C-5′), 17.9 (C-6′), 171.9 (C-1″), 43.4 (C-2″), 26.7 (C-3″), 16.4 (C-4″) and 11.5 (C-5″).

### Chemical structure of shikonin derivatives

**Figure 1.**
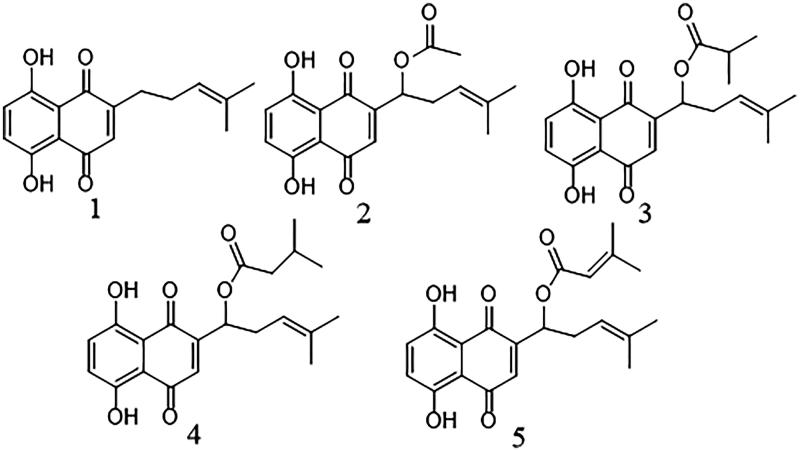
Chemical structure of shikonin derivatives. **1**: deoxyshikonin; **2**: acetylshikonin; **3**: isobutyrylshikonin; **4**: β,β′-dimethylacrylshikonin; **5**: isovalerylshikonin.

**Figure 2.**
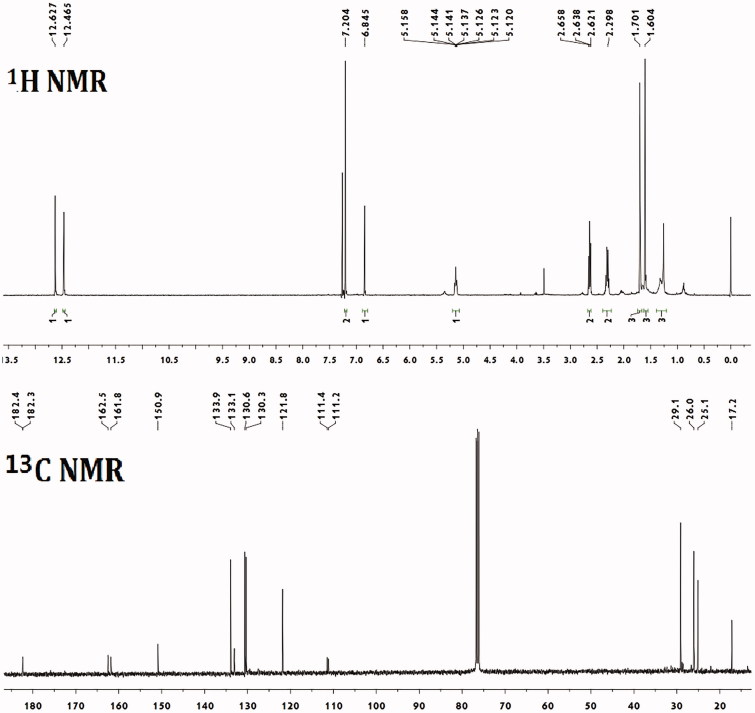
The ^1^H and ^13^C NMR chromatograms of shikonin derivative deoxyshikonin occurring in *Arnebia euchroma*.

Five naphthoquinones (Figure 1) were isolated from the extracts of *A. euchroma* by bioassay-guided fractionation. These shikonin derivatives were identified as deoxyshikonin (**1**) (Figure 2) (Ozgen et al. 2004), acetylshikonin (**2**) (Ko et al. 1995), isobutyrylshikonin (**3**) (Cui et al. 2008), β,β′-dimethylacrylshikonin (**4**) (Hu et al. 2006) and isovalerylshikonin (**5**) (Ko et al. 1995), using spectral analysis by ^1^H and ^13^C NMR and comparison with literature data.

### Effects of shikonin derivatives on the proliferation of colonic cancer cells

**Figure 3.**
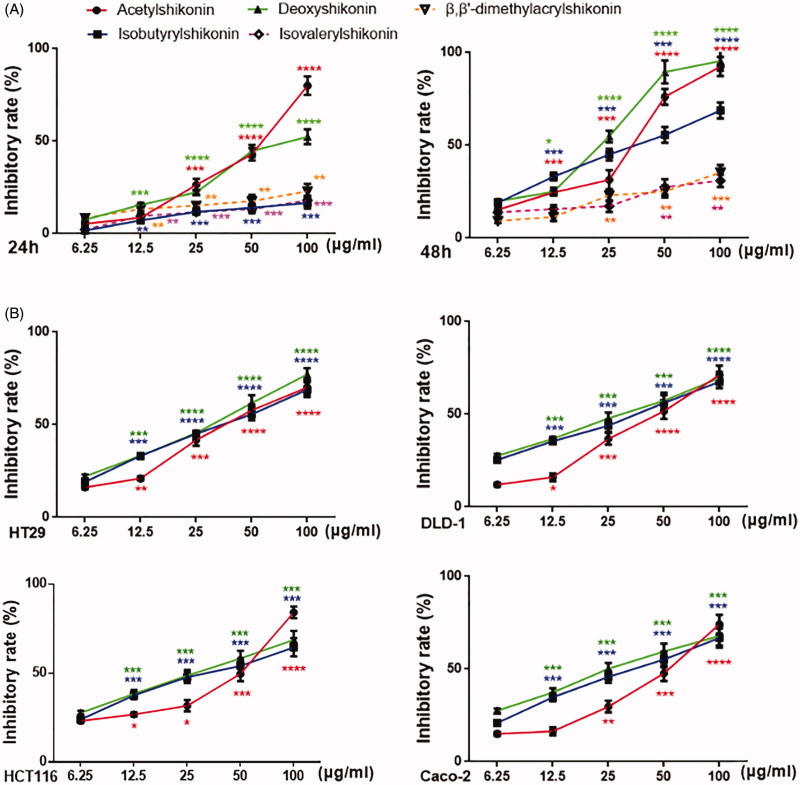
Effects of shikonin derivatives on the cell inhibitory of human colonic cancer cell lines *in vitro*. (A) Shikonin derivatives (0, 6.25, 12.5, 25, 50 and 100 μg/mL) inhibited the growth of HT29 cells for 24 or 48 h, and the cells inhibitory was assessed by MTT assay; (B) Caco-2, HCT116, DLD-1 and HT29 cells were treated with acetylshikonin, isobutyrylshikonin and deoxyshikonin at different concentrations (0, 6.25, 12.5, 25, 50 and 100 μg/mL) for 48 h, followed by analysis with CCK-8 assay. Results were obtained from three independent experiments, and the dots represent mean ± SD.

To examine the antiproliferative effects of shikonin derivatives, first, we examined cell viabilities assayed by MTT method. Compared to the untreated cells, the treatments with these shikonin derivatives showed significant inhibition on the growth of HT29 cells. Moreover, deoxyshikonin, acetylshikonin and isobutyrylshikonin had obvious effects, especially after 48 h of treatment (Figure 3(A)). Interestingly, the inhibition rates of cell viability with deoxyshikonin treatment rose up accompanied with the increase of concentration at 24 h, although lower than acetylshikonin at the maximum concentration of 100 μg/mL. However, it showed the best inhibition rate at 48 h. At the same time, we calculated the IC_50_ values of five shikonin derivatives against HT29 cells (Table 1). IC_50_ values of deoxyshikonin treatment were 31.00 ± 0.78 μM at 24 h, while 10.97 ± 1.23 μM at 48 h. Second, to further verify the significant inhibitory effect of deoxyshikonin on CRC, we also adopted CCK-8 assay. Consistent with the results of MTT assay, deoxyshikonin at low concentration inhibited the growth of other human colonic cancer cells including DLD-1, HCT-116, Caco-2 and HT29 cells comparing with non-treated cells. Most importantly, deoxyshikonin had an antiproliferative effect with a steady increase of inhibitory rate on HT29 cells at 48 h. Based on these results, deoxyshikonin might exhibit dose-dependent inhibitory effects on colonic cancer cells at 24 and 48 h, especially after 48 h exposure (Figure 3(B)).

**Table 1.** Antiproliferative activities of shikonin derivatives against HT29 cells.

Shikonin derivatives	IC_50_ at 24 h (μM)	IC_50_ at 48 h (μM)
Acetylshikonin	18.43 ± 0.68	9.33 ± 0.98
Isobutyrylshikonin	59.61 ± 0.38	14.00 ± 0.25
Deoxyshikonin	31.00 ± 0.78	10.97 ± 1.23
β,β′-Dimethylacrylshikonin	62.72 ± 0.57	36.02 ± 0.86
Isovalerylshikonin	58.31 ± 0.57	44.81 ± 0.96

### Effects of deoxyshikonin on HT29 cells apoptosis

**Figure 4.**
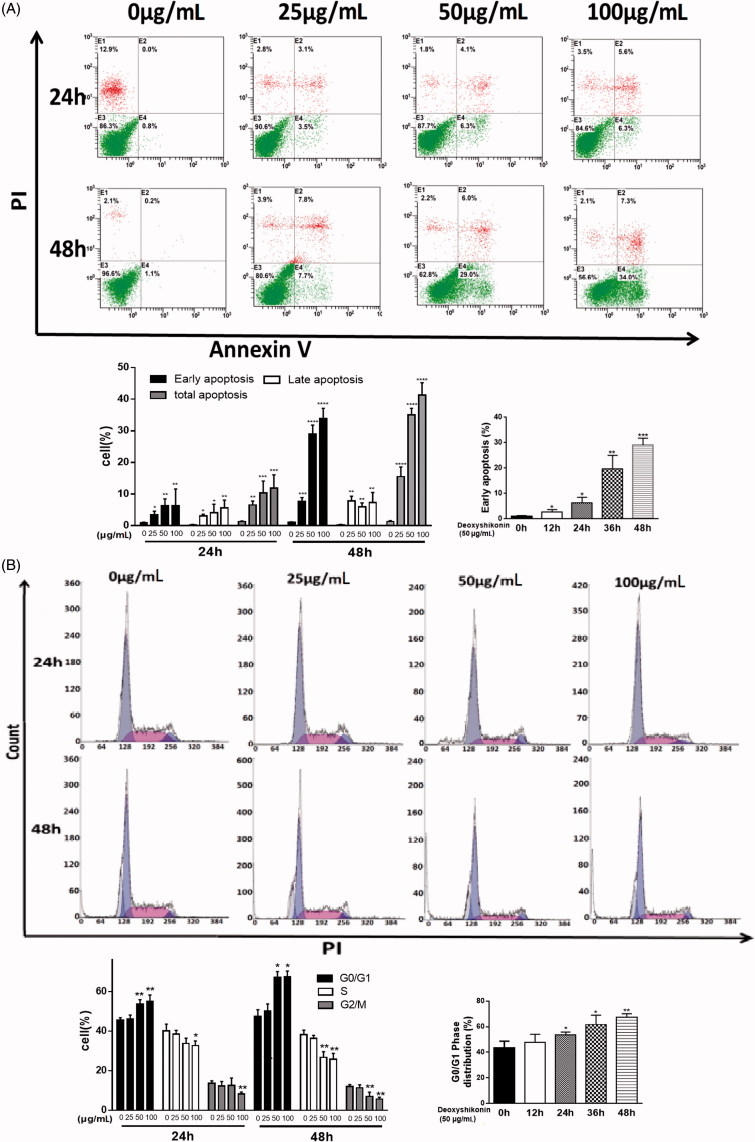
Effects of deoxyshikonin on apoptosis and cell cycle distribution in HT29 cells. (A) Apoptotic cells were detected by flow cytometry. Histograms demonstrate percentages of apoptotic cells in culture. Early apoptotic cells of 50 μg/mL deoxyshikonin-treatment for the indicated time periods were processed statistically. (B) Cell cycle distribution was detected by flow cytometry. Histograms demonstrate the percentage of tumour cells at different phases of the cell cycle. (C) G0/G1 phase distribution of 50 μg/mL deoxyshikonin-treatment for the indicated time periods were processed statistically. Data were represented as mean ± SD form at least three independent experiments. *p < 0.05, **p < 0.001, ***p < 0.0001, ****p < 0.00001 versus control (0 μg/mL) or control (0 h).

To determine whether deoxyshikonin can induce apoptotic death in HT29 cells, flow cytometry was performed by Annexin V and PI staining. At 24 h, the percentage of early apoptotic cells was 6- to 9-fold higher in HT29 cells treated with 50 μg/mL deoxyshikonin than that in non-treated cells. Moreover, the ratio of early apoptotic cells increased from 1% to 29% in a dose-dependent manner by being treated with 0–50 μg/mL deoxyshikonin at 48 h. Meanwhile, the early stages of apoptosis changes were occurred in the period from 0 to 48 h (Figure 4(A)). Together, these data indicate that deoxyshikonin may induce early apoptotic cells death (*p < 0.05, **p < 0.001, ***p < 0.0001, ****p < 0.00001 versus control (0 μg/mL) or control (0 h)).

### Effect of deoxyshikonin on the cell cycle arrest

Compared with non-treated cells, deoxyshikonin treatment led to a dose-dependent increase in the percentage of cells at G0/G1 phase. It is worth pointing out that the percentage of G0/G1 cells increased from approximately 44% to 67% in HT29 cells after treatment with 0–50 μg/mL deoxyshikonin at 48 h, accompanied by a significant decrease in the percentage of cells at S and G2/M phases, but at a higher concentration, deoxyshikonin (100 μg/mL) resulted in no change compared to 50 μg/mL deoxyshikonin. Furthermore, in cells treated with 50 μg/mL deoxyshikonin, the accumulation of cells in the G0/G1 phase was associated primarily with time (from 0 to 48 h). Together, these results indicated that the growth-suppressive effect of deoxyshikonin was partly due to a G0/G1-phase arrest (Figure 4(B)) (*p < 0.05, **p < 0.001, ***p < 0.0001, ****p < 0.00001 versus control (0 μg/mL) or control (0 h)).

### Inhibitory effects of deoxyshikonin on PI3K/Akt/mTOR pathway

**Figure 5.**
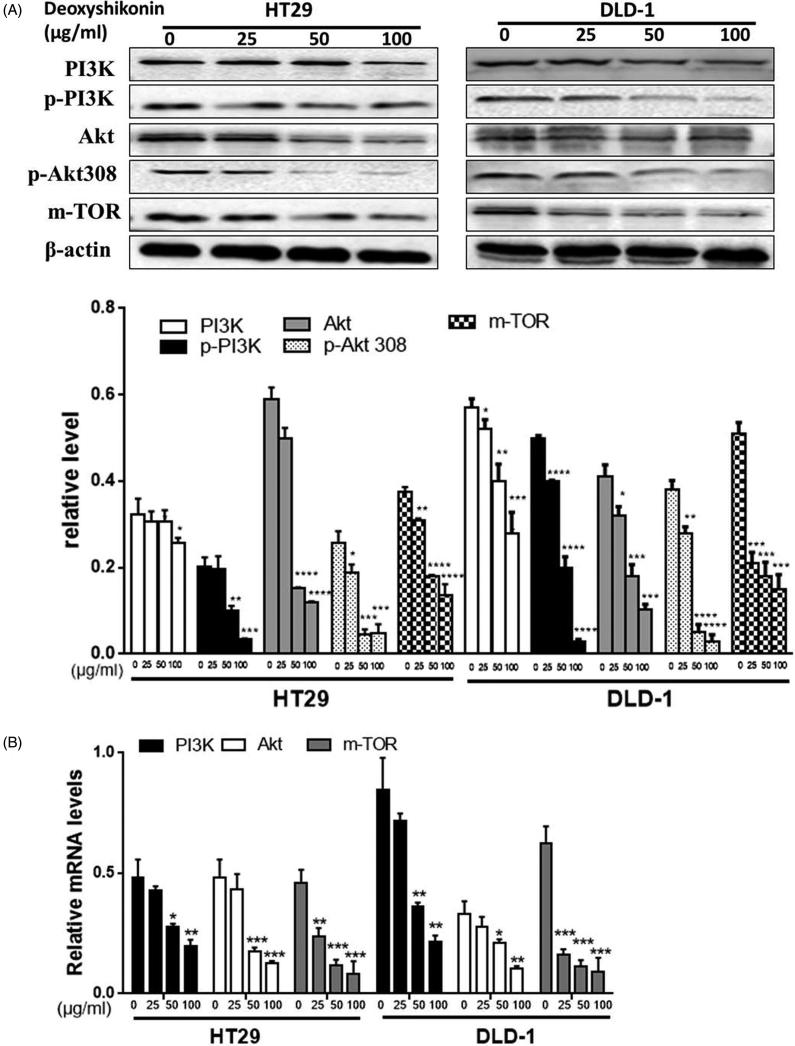
Effects of deoxyshikonin on Akt/PI3K/mTOR signalling pathway in HT29 cells. (A) PI3K, *p*-PI3K, Akt, *p*-Akt308 and mTOR proteins in HT29 and DLD-1 cell lines were determined with specific antibodies. β-Actin was used as loading control. The relative intensity of each protein was normalized with β-actin. (B) The expression levels of Akt, PI3K, mTOR mRNA in HT29 and DLD-1 cell lines were measured by RT-PCR. Data were results of three independent experiments with mean ± SD. *p < 0.05, **p < 0.001, ***p < 0.0001 and ****p < 0.00001 versus control (0 μg/mL).

**Figure 6.**
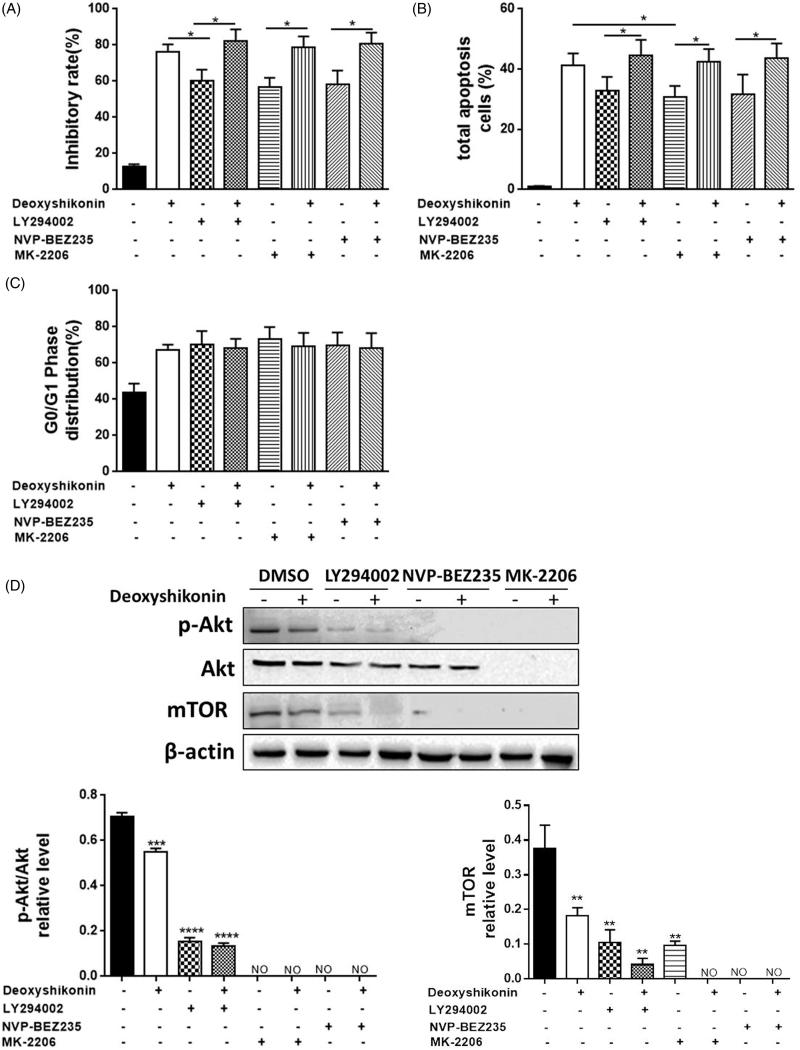
Deoxyshikonin mediated HT29 cells viability, cell apoptosis and cycle arrest depended on the PI3K/Akt/mTOR signalling pathway. HT29 cells were pretreated with LY294002, or NVP-BEZ235, or MK-2206, then co-treated with 50 μg/mL deoxyshikonin for further 48 h. (A) Cell viability was assessed by MTT assay; (B) cell apoptosis and cell cycle arrest were detected by flow cytometry; (C) Akt and *p*-Akt308 proteins were determined by Western blotting. Data were presented as mean ± SD of three independent tests. *p < 0.05, **p < 0.001, ***p < 0.0001 and ****p < 0.00001 versus control (0 μg/mL).

Since we have observed that deoxyshikonin could induce apoptosis in HT29 cells, we further investigated its possible mechanisms. PI3K/Akt/mTOR signalling pathway is important to regulate proliferation, differentiation and migration of cancer cells. As shown in Figure 5(A), treatment with deoxyshikonin (0, 25, 50 and 100 μg/mL) for 48 h exerted a decrease of PI3K, *p*-PI3K, Akt, *p*-Akt308 and mTOR proteins expression in HT29 and DLD-1 cell lines. Similarly, the relative mRNA levels of PI3K/Akt/mTOR (Figure 5(B)) also reduced in HT29 and DLD-1 cell lines. To further determine the relationship between cell apoptosis and PI3K/Akt/mTOR pathway, LY294002 (a PI3K inhibitor), NVP-BEZ235 (a PI3K and mTOR inhibitor) and MK-2206 (an Akt inhibitor) were exposed to deoxyshikonin for 48 h, respectively. HT29 cells treated with these above inhibitors plus 50 μg/mL deoxyshikonin had higher rates of cell inhibitory and total apoptosis cells than that of being treated with deoxyshikonin alone, and there was little change observed in the G0/G1 phase distribution (Figure 6(A–C)). Similarly, deoxyshikonin plus LY294002 treatment caused a significant decrease in the ratio of *p*-Akt308/Akt, comparing with deoxyshikonin alone (Figure 6(D)). These findings suggested that deoxyshikonin inhibited proliferation and promoted apoptosis of CRC through suppressing the PI3K/Akt/mTOR signalling pathway (*p < 0.05, **p < 0.001, ***p < 0.0001 versus control (0 μg/mL)).

### Deoxyshikonin exhibited antitumour activity *in vivo* via PI3K/Akt/mTOR pathway

**Figure 7.**
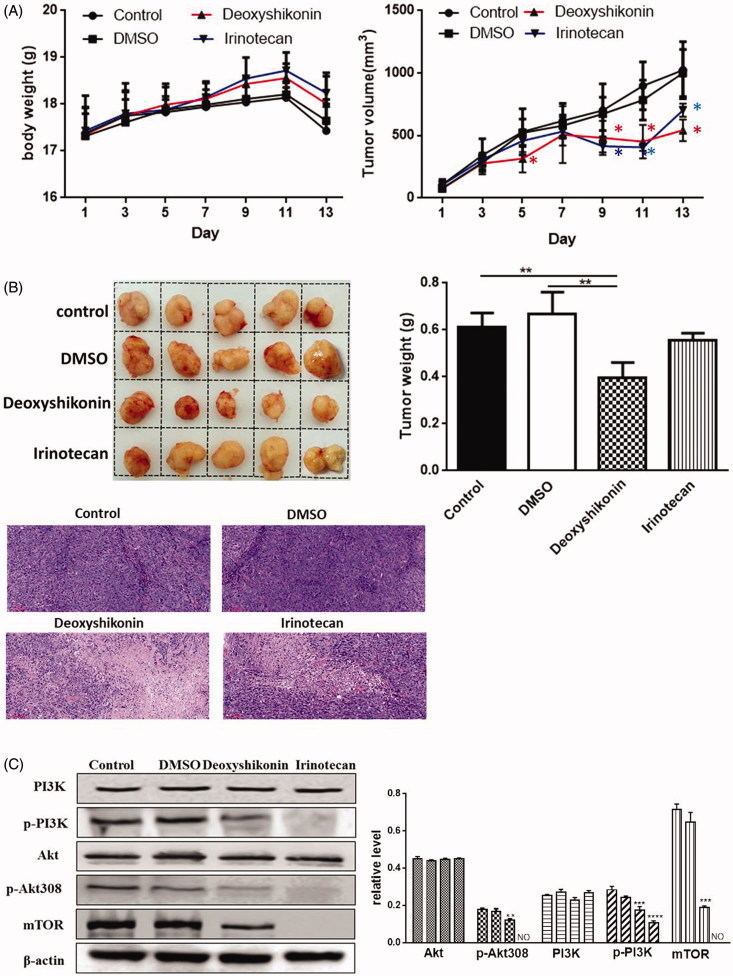
Anticancer activity of deoxyshikonin *in vivo* via PI3K/Akt/mTOR pathway. BALB/c nude mice were injected with DLD-1 cells into the subcutaneous tissue of the right auxiliary region. Xenograft model was established when tumours reached an average size of 62.5 mm^3^, and the treatments were given different drugs by intraperitoneal injection for a total of 13 days: control, DMSO (1% DMSO, every two days), deoxyshikonin (20 mg/kg in 1% DMSO, every two days), irinotecan (66.7 mg/kg, every four days). (A) Body weights and tumour volumes were measured once daily; (B) images, tumour weights and H&E staining (200× magnification) were measured after 13 days of treatment; (C) the proteins of tumour tissues were blotted with antibodies against indicated proteins (PI3K, p-PI3K, Akt, p-Akt308 and mTOR), and β-actin was blotted as a loading control. Data are presented as mean ± SD. *p < 0.05, **p < 0.001, ***p < 0.0001 and ****p < 0.00001 versus control.

To analyse the antitumour effects of deoxyshikonin, we used a xenograft tumour model by transplanting DLD-1 cells to nude mice. Compared with the control group, treatment with 20 mg/kg deoxyshikonin markedly suppressed the growth of xenograft tumours on day 5, 9 and 11, while there were no significant changes in body weight of the mice (Figure 7(A)). At the end of the study, we removed and weighed the tumours. The weight of the tumour tissues from mice treated with deoxyshikonin was lighter than that of the control group. In addition, tumour tissues from mice treated with deoxyshikonin had large areas of continuous necrosis than those that received the control treatment by H&E staining. However, tissue necrosis interspersed with viable cancer cells were detected in the untreated control tumours (Figure 7(B)). Western blotting analysis from representative tumour tissues demonstrated that deoxyshikonin decreased PI3K, *p*-PI3K, Akt, *p*-Akt308 and mTOR proteins expression, which were consistent with the *in vitro* results (Figure 7(C)) (*p < 0.05, **p < 0.001, ***p < 0.0001 versus control (0 μg/mL)).

## Discussion

Shikonin, a natural naphthoquinone, has antitumour and anti-inflammation activities. However, it is rare that oral shikonin is directly administered in the clinic because of its toxicity in the body (Tang et al. 2017). Recent studies on the extraction, synthesis and biological activity of high-performance but nontoxic shikonin derivatives are receiving increasing attention (Su et al. 2014; Lu et al. 2015). In this study, we verified the antitumour activity and mechanism of five shikonin derivatives from the roots of *A. euchroma*, acetylshikonin, isobutyrylshikonin, deoxyshikonin, β,β′-dimethylacrylshikonin and isovalerylshikonin. Among these shikonin derivatives, we found deoxyshikonin significantly inhibited the growth of human colon cancer cells. A reduction in cell viability, inhibition of cell proliferation, induction of apoptosis, and arrested cell cycle were exhibited in deoxyshikonin-treated cells. Furthermore, deoxyshikonin suppressed CRC through PI3K/Akt/mTOR signalling pathway.

A key character of cancer progression is cell proliferation (Hanahan and Weinberg, 2011). Based on the results of MTT analysis, shikonin derivatives showed different degree on the proliferation of human colon cancer cells, with the experimental dose range of 1.69–36.76 μM. But the effects of β,β′-dimethylacrylshikonin and isovalerylshikonin were very weak. Simultaneously, using CCK-8 analysis on human colon DLD-1, HT29, HCT116 and Caco-2 cancer cells, the remaining three drugs dramatically inhibited their proliferation. Importantly, we found deoxyshikonin dramatically and smoothly reduced the rate of inhibitory of HT29 cells, which performed the most effective at 48 h. Meanwhile, cell proliferation was in a dose-dependent manner and IC_50_ value was 10.97 ± 1.23 μM at 48 h. Hence, deoxyshikonin might become a new candidate compound against CRC and it was necessary to understand the mechanism of anticancer action.

Excessive tumour cell growth leads to tumour progression, as well as abnormal apoptosis and cell cycle arrest. At present, antitumour drugs used in the clinic mainly induce tumour cell necrosis and apoptosis (Li et al. 2012). Owing to the obviously inhibition of HT29 cell proliferation by deoxyshikonin, we speculated that it might modulate the apoptosis and cell-cycle progression. Comparing with untreated cells, the total apoptosis rate of HT29 cells treated with deoxyshikonin were significantly increased in a dose-dependent manner, consistent with the results of the previous proliferation experiments. Moreover, the most significant effect occurred at 48 h, especially in the early stages of apoptosis. However, deoxyshikonin induced a reduction in S phase or G2/M population phase population at high concentration (18.39–36.76 μM) by cell-cycle distribution analysis, which was associated with the accumulation of cells in the G0/G1 phase. Meanwhile, these results were also monitored the effect of deoxyshikonin on G0/G1 phase at different time points at different time points. Together, these results indicated that apoptosis and cell cycle arrest might be mainly responsible for deoxyshikonin-induced colon cancer cells growth inhibition.

Previous studies have indicated that the PI3K/Akt/mTOR signalling pathway plays an essential role in tumour, including regulating tumour cell proliferation, promoting tumour cell migration and invasion, affecting cell cycle progression, participating in neovascularization (Popolo et al. 2017; Woo et al. 2017; Zhang et al. 2017). PI3K activates and phosphorylates the key downstream kinases-Akt, then triggers mTOR through a series of regulators (Kumar et al. 2014; Qian et al. 2017). The signal cascade is the main pathway to inhibit the apoptosis of tumour cells. In addition, a loss of PI3K/Akt/mTOR expression was associated with more-advanced clinicopathological features and an inferior colon cancer prognosis (Vanhaesebroeck et al. 2012; Green et al. 2013; Rodon et al. 2013; Yu et al. 2017). Inhibition of this pathway has been suggested to account for anticancer effects elicited by various drugs (He et al. 2014; Pang et al. 2014). This study aimed to assess the effects of deoxyshikonin related to PI3K/Akt/mTOR pathway. Interestingly, untreated HT29 cells had the high expressions of Akt, PI3K and mTOR, while deoxyshikonin reduced their expressions and at the same time reduces the levels of *p*-PI3K and p-Akt. Moreover, the addition of PI3K/Akt/mTOR inhibitors reduced *p*-Akt308/Akt ratio (Figures 5 and 6). Inhibition of PI3K/Akt/mTOR signalling pathway by co-culturing with their specific inhibitors and deoxyshikonin caused higher inhibitory rate, total apoptosis cells and the ratio of G0/G1 phase distribution. Our findings indicated that *p*-Akt308 is a prerequisite for the antitumour effect of deoxyshikonin and deoxyshikonin could promote cell apoptosis mainly by inhibiting PI3K/Akt/mTOR pathway.

Meanwhile, similar results were observed *in vivo*. We found that 20 mg/kg deoxyshikonin efficiently inhibited the volume and weight of tumour in DLD-1 xenograft nude mice. Interestingly, the mice treated with both deoxyshikonin and irinotecan exhibited almost no body weight change, suggesting that they showed little toxicity *in vivo*. In accordance with the H&E staining results, the most effective suppression of tumour growth in deoxyshikonin group was attributable to cell apoptosis. Based on Western blot analysis, the antitumour effect of deoxyshikonin might inhibit PI3K/Akt/mTOR pathway, which was consistent with *in vitro* results.

However, we must also pay attention to the limitations of this study. First, other signals may also be involved in the anticancer effect of deoxyshikonin in CRC, because over-expressed Akt is not completely reversed, or even that the dose is not enough to suppress the total expression of Akt. Second, the lack of phosphorylation of m-TOR, a downstream factor of the PI3K/Akt pathway, was discussed. Finally, animal experiments are not considered fully and just only used as a preliminary study on the effect and mechanism of deoxyshikonin anti-CRC.

In conclusion, this research presumed that deoxyshikonin inhibited the proliferation and apoptosis of colon cancer via down-regulating PI3K/Akt/mTOR partway. Therefore, deoxyshikonin might be a potential therapeutic drug to control the growth of colon cancer. However, the promising anticancer mechanism of deoxyshikonin should be further studied for successful therapy of human colon cancer and further *in vivo* pharmacological and clinical investigations are required.
